# A Peculiar Cause for Metabolic Acidosis in the Newborn

**Published:** 2012-10-01

**Authors:** Sundeep Kisku, Sudipta Sen, Reju Joseph Thomas, Arindam Dastidar, Niranjan Thomas

**Affiliations:** Department of Pediatric Surgery, Christian Medical College, Vellore, Tamil Nadu.; 1 Department of Neonatology, Christian Medical College, Vellore, Tamil Nadu.

**Keywords:** Ileal duplication, Gastric heterotopia, Perforation, Metabolic acidosis

## Abstract

Metabolic acidosis is often encountered in a sick neonate and intestinal duplication with heterotopic gastric mucosa is a well-established condition. We present a previously unreported relationship between neonatal metabolic acidosis, resulting from transperitoneal absorption of hydrochloric acid, and a ruptured non- communicating ileal duplication cyst with gastric mucosal heterotopia. The neonate recovered rapidly after resection of the ileal duplication. We present this case to highlight a rare but surgically correctable cause of neonatal metabolic acidosis.

## INTRODUCTION

Causes of metabolic acidosis in the neonatal period include birth asphyxia, sepsis, cold stress, dehydration, congenital heart diseases (hypoplastic left heart syndrome, coarctation), renal disorders (polycystic kidneys, renal tubular acidosis) and inborn errors of metabolism. Necrotizing enterocolitis, bowel perforation and gangrene are some common surgical causes of neonatal metabolic acidosis.

Intestinal duplications are uncommon congenital lesions that can occur anywhere from the mouth to the anus with an incidence of 1 in 4,500 autopsies [1]. The small bowel, particularly ileum (30% -50%) is the most common site of duplication cysts [2]. The clinical presentation is varied and includes obstructive symptoms (caused by mass effect) in a non- communicating duplication cyst or peptic erosion with gastro-intestinal bleed in a communicating duplication. A ruptured non-communicating, acid secreting ileal duplication cyst presenting with metabolic acidosis has not been reported, to the best of authors' knowledge.

## CASE REPORT

A nine hour old term female newborn born by normal vaginal delivery presented to the emergency department with abdominal distention and respiratory distress since birth. There was no antenatal history of maternal infections or birth asphyxia. The child had passed urine and meconium within an hour after birth. On examination the child was tachypneic (respiratory rate 80/min). The heart rate was 150/min and the digital peripheral oxygen saturation was (Sp02)88% on room air. The capillary refill was prolonged (> 3 sec). Normal breath sounds were heard bilaterally. The abdomen was distended and had a bluish hue. A presumptive diagnosis of sepsis was considered and the child was administered IV fluids, O2 by nasal prongs and IV antibiotics (Crystalline Penicillin, Gentamycin and Metronidazole) after a blood culture. Inotropic (Dopamine) support was initiated. Blood gas revealed partially compensated metabolic acidosis with hyperchloremia and a normal anion gap.{ pH 7.32, Hco3 7.7, mmol/L, PCO2 15 .3mmHg , Acid Base Excess -16.2, Chloride 116 mmol/L(normal 95-105 mmol/L), 

Hemoglobin 18.1g%,total count 27,424 cell/cc (polymorphs 85%, lymphocytes 15%), serum sodium 125 mmol/L, potassium 5.0 mmol/L, anion gap 6.3 (normal 5-15)}. The CRP was 14.28 mg/L. The neonate continued to be tachypneic with irregular respiration and was intubated and ventilated.

**Figure:**
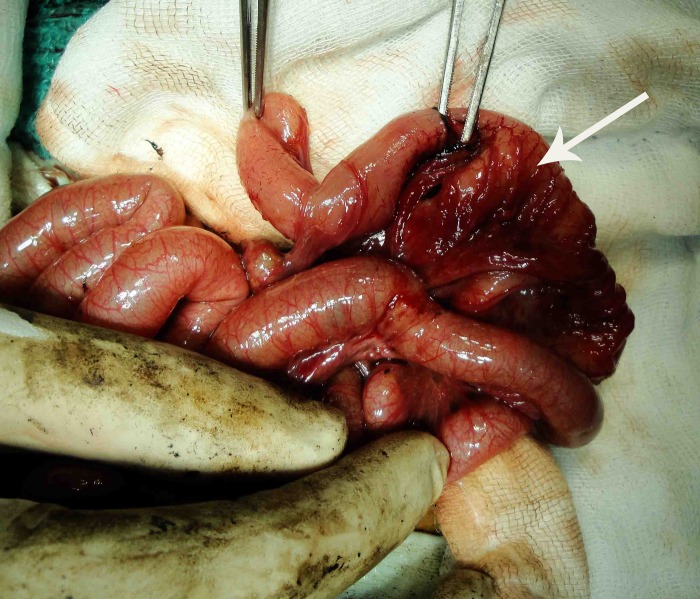
Figure 1: Perforated ileal duplication.

**Figure:**
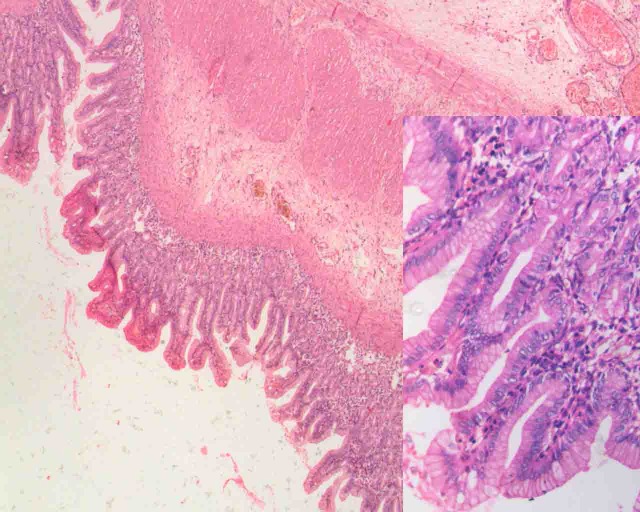
Figure 2: Histopathology showing ectopic gastric mucosa in the ileal duplication.

The neonate was passing normal meconium and plain X-ray (supine and lateral decubitus) of the abdomen revealed non- dilated gas filled bowel loops indicating that intestinal obstruction was unlikely. Ultrasonogram of the abdomen reported ascites and on aspiration, hemorrhagic fluid was obtained; biochemical analysis detected acid hematin (5gm% at pH of 7.0). Serial blood lactate levels showed a rise from 2.5 to 3.1mmol/L. A provisional diagnosis of bowel gangrene was considered and laparotomy performed under general anesthesia. The peritoneal cavity was filled with copious hemorrhagic fluid. There was a non-communicating 10cm long ileal duplication cyst that was perforated (Figure 1). The proximal end of the cyst was closely adherent to the native ileum and the distal end was free. The distal end of the duplicated cyst was excised stripping the mucosa off the proximal segment that had a common wall with the native ileum. Histology revealed the presence of gastric mucosal heterotopia in the ileal duplication cyst (Figure 2). The child displayed prompt and dramatic postoperative resolution of the metabolic acidosis.{pH 7.39, Hco3 22.5 mmol/L, PCO2 38 mmHg, Base Excess -2.1, Chloride 107 mmol/L}. The child made a full recovery and was discharged on day 10 and has completed 2 yrs of uneventful follow up.

## DISCUSSION

Most small bowel duplications are non-communicating and about 50% may contain ectopic gastric mucosa; around 10% can have more than one heterotopic mucosa. One third of the small bowel duplication cysts are symptomatic in the neonatal period and more than 3/4ths of patients clinically manifest before 2 years of age [3,4,5]. 

Ileal duplication cysts present with acute abdominal pain, bowel obstruction, intussusception, palpable mass, hemorrhage, and perforation [4]. Many are detected antenatally and remain asymptomatic. Acute presentations of ileal duplication cysts in newborn are rare. There have been presentations with severe respiratory distress [6] and bowel obstruction [7], atresia [8] and volvulus [9].

This neonate presented with shock and metabolic acidosis; the most common differential diagnosis for this being sepsis. In this case, sepsis was unlikely due to the presence of hyperchloremic metabolic acidosis with a normal anion gap, a negative blood culture and sepsis screen (WBC count and CRP). The ruptured non- communicating ileal duplication cyst contained gastric mucosal heterotopia and acid hematin was detected in the acidic hemorrhagic peritoneal fluid (peritoneal fluid pH 7.0, blood gas pH 7.39). Acid hematin is the acid salt of HCl acting on hemoglobin [10]. A prompt and dramatic recovery after excision of the duplication cyst suggests a relationship. 

It is probable that the acid (HCl) secreted by the heterotopic gastric mucosa distended and ulcerated with subsequent perforation of the non-communicating ileal duplication. The resultant bleeding (hemoglobin within RBCs) from the perforated wall reacted with HCl to form acid hematin. The efflux of HCl into the peritoneal cavity led to its absorption into the blood stream resulting in hyperchloremic metabolic acidosis. Other causes of normal anion-gap hyper-chloremic metabolic acidosis in the newborn include conditions like renal immaturity, bicarbonate losses from renal sources (renal tubular acidosis, uremia, obstructive uropathy, mineralocorticoid deficiency), diarrhea, infusion chloride containing salts, acetazolamide therapy and pancreatic and urinary fistulae. These factors were not present in the neonate reported herein, and this case demonstrates a rare but surgically amenable cause of neonatal metabolic acidosis.

Definitive treatment of ileal duplication cysts requires complete surgical excision, when feasible. A segmental resection of the involved bowel is usually required, because enteric duplication cysts are always mesenteric and share their blood supply and muscular wall with the adjacent bowel. The healthy normal bowel may be preserved by mucosal stripping of the duplication cyst.

## Footnotes

**Source of Support:** Nil

**Conflict of Interest:** None
